# Effect of Chitosan on the Activity of Water-Soluble and Hydrophobic Porphyrin Photosensitizers Solubilized by Amphiphilic Polymers

**DOI:** 10.3390/polym13071007

**Published:** 2021-03-25

**Authors:** Valeriya V. Kardumyan, Nadejda A. Aksenova, Victoria A. Timofeeva, Alexey V. Krivandin, Olga V. Shatalova, Alexander S. Dubovik, Irina G. Plashchina, Peter S. Timashev, Anna B. Solovieva

**Affiliations:** 1 N.N. Semenov Federal Research Center for Chemical Physics, Russian Academy of Sciences, Kosygin St. 4, Moscow 119991, Russia; naksenova@mail.ru (N.A.A.); vik.timofeeva@gmail.com (V.A.T.); timashev.peter@gmail.com (P.S.T.); ann.solovieva@gmail.com (A.B.S.); 2 Institute for Regenerative Medicine, Sechenov First Moscow State Medical University (Sechenov University), 8-2 Trubetskayast, Moscow 119991, Russia; 3 N.M. Emanuel Institute of Biochemical Physics, Russian Academy of Sciences, Kosygin St. 4, Moscow 119334, Russia; a.krivandin@sky.chph.ras.ru (A.V.K.); shatalova@sky.chph.ras.ru (O.V.S.); adubovik@ineos.ac.ru (A.S.D.); igplashchina@yahoo.com (I.G.P.); 4 A.N. Nesmeyanov Institute of Organoelement Compounds, Russian Academy of Sciences, Vavilov St. 28, Moscow 119991, Russia; 5 Chemistry Department, M.V. Lomonosov Moscow State University, Vorobyevy Gory, Moscow 119334, Russia

**Keywords:** chitosan, porphyrin, Pluronic F127, polyvinylpyrrolidone, tryptophan photo-oxidation

## Abstract

In this work, we studied the photocatalytic activity of photosensitizers (PSs) of various natures solubilized with polyvinylpyrrolidone (PVP) and ternary block copolymer ethylene and propylene oxide Pluronic F127 (F127) in a model reaction of tryptophan photo-oxidation in water in the presence of chitosan (CT). Water-soluble compounds (dimegin and trisodium salt of chlorin e6 (Ce6)) and hydrophobic porphyrins (tetraphenylporphyrin (TPP) and its fluorine derivative (TPPF20)) were used as PSs. It was shown that the use of chitosan (Mw ~100 kDa) makes it possible to obtain a system whose activity is comparable to that of the photosensitizer-amphiphilic polymer systems. Thus, the previously observed drop in the photosensitizing activity of PS in the presence of a polysaccharide and amphiphilic polymers (AP) was absent in this case. At the same time, chitosan had practically no inhibitory effect on hydrophobic porphyrins solubilized by Pluronic F127.

## 1. Introduction

Due to the increasing resistance of bacterial and fungal pathogens to drug therapy, alternative methods of therapy have become very important for the treatment of local infections. Antimicrobial photodynamic therapy (aPDT) may be one of these methods. In most cases, aPDTis used in the treatment of superficial injuries of the skin and soft tissues (purulent wounds, burns, trophic ulcers). As a result of such therapy, complete destruction of bacterial cells without the development of resistance to aPDT occurs [1]. Photosensitizers (PS), generating singlet oxygen(^1^O_2_)—an active oxidizing agent—upon excitation with light, are successfully used in photodynamic therapy (PDT) of diseases of various etiologies [2,3,4]. In the clinical practice of photodynamic therapy in Russia, mainly drugs based on water-soluble phthalocyanines (photosens [5]) and natural porphyrins (photohem [5]), chlorins (photolon [6], photoditazin [7]) and synthetic porphyrin (temoporfin [8]) are used as PS. In the practice of aPDT, cationic photosensitizers [3] proved to be the most effective, and the “cationic” fragment is often represented by a polymer. In particular, Nitzan et al. [9] developed a polymeric PS, consisting of deuteroporphyrin and polycationic peptide polymyxin B, and Strakhovskaya et al. [10] used a conjugate of anionic dyes (phthalocyanines) with cationic polylysine. The biologically active polysaccharide chitosan (CT) is also considered as a “cationic” component.

In the most frequently used photosensitizers, the maximum absorption is realized in the Soret band in the wavelength range λ = 400–430 nm characteristic for such compounds. However, PS used in PDT for the treatment of internal diseases of various origins should have a pronounced absorption, preferably with a high extinction coefficient (ε) in the red part of the visible spectrum (the so-called “transparency window”) in the range of 600–800 nm, where the light penetration into the tissues is maximized [11].

One of the advantages of aPDT, when it comes to the treatment of superficial injuries, is the ability to excite PS with light corresponding to the Soret band (λ ~ 400 nm) with maximum absorption. In this case, synthetic PPS–tetraphenylporphyrin (TPP) and its meso derivatives, including the fluorine-containing ones which do not have intense absorption bands in the red region of the spectrum, are in demand. They have a high quantum yield of singlet oxygen photogeneration (ΦΔ ~0.6–0.8) and are relatively simple to synthesize. Interest in fluorine-substituted tetraphenylporphyrins is due to both the high quantum yield (ΦΔ) of generation of ^1^O_2_ and increased photostability, compared with unsubstituted PSs [12].

Currently, to increase the effectiveness of treatment of local infections (hard-to-heal wounds, complicated burns), it is customary to combine different methods of treatment, in particular to enhance aPDT through the use of additional biologically active compounds: proteolytic enzymes (lidase [13]), antioxidants [14], and biomolecules with bactericidal properties. A biologically active polysaccharide, chitosan, can be used as a bactericidal agent. Chitosan is a polycationic linear polysaccharide, soluble in weakly acidic media; chitosan-based compounds are used in the treatment of wounds and burns [15]. The cationic nature of chitosan determines its ability to interact with synthetic or natural polymers containing negative charges. This property allows it to easily bind to the outer side of the cell membranes of living organisms (proteins, glycoproteins, negatively charged phospholipids) [10]. It is possible that chitosan interacting with the cell membrane can change its permeability [10,15,16], which leads to disintegration of the outer membrane and an increase in its permeability for dyes. In addition, it is believed that such interaction of chitosan with the bacterial cell membrane leads to membrane destruction and subsequent leakage of protein and other intracellular components, i.e., to cell death [15,16,17]. The properties of chitosan (both biological and physicochemical) are determined by its molecular weight (Mw), the quantitative ratio of acetylated and deacetylated units, as well as the nature of their arrangement along the polymer chain [18]. These features of chitosan determine the variety of its properties, including the bactericidal and fungicidal ones.

We have previously shown that, upon solubilization of hydrophobic and water-soluble porphyrins by amphiphilic polymers, an interaction between PS and AP occurs due to the formation of hydrogen and hydrophobic bonds, which leads to the disaggregation of PSs and an increase in their photocatalytic activity by 1.2–1.5 times [19].

Such PS-AP complexes containing chitosan (60 kDa) showed a stable antibacterial effect during aPDT of infected wounds in model animals (rats) [20]. However, as previously shown, the photosensitizing activity of PSs in the generation of singlet oxygen decreases in the presence of chitosan [21], since water-soluble anionic PSs interact with polycationic chitosan (20 kDa), which leads to their aggregation [22]. In the presence of AP, the activity of the PS-AP-CT ternary systems did not exceed the activity of pure porphyrin, and there was practically no further increase in the activity of PS in the complex with AP.

In this work, it is shown that when using chitosan with Mw ~100 kDa, it is possible to obtain photosensitizing porphyrin-containing polymer-polymer systems (PS-AP-CT_100_), the activity of which in the generation of singlet oxygen exceeds the activity of binary PS-AP systems.

## 2. Materials and Methods

### 2.1. Reagents

**Figure 1:**
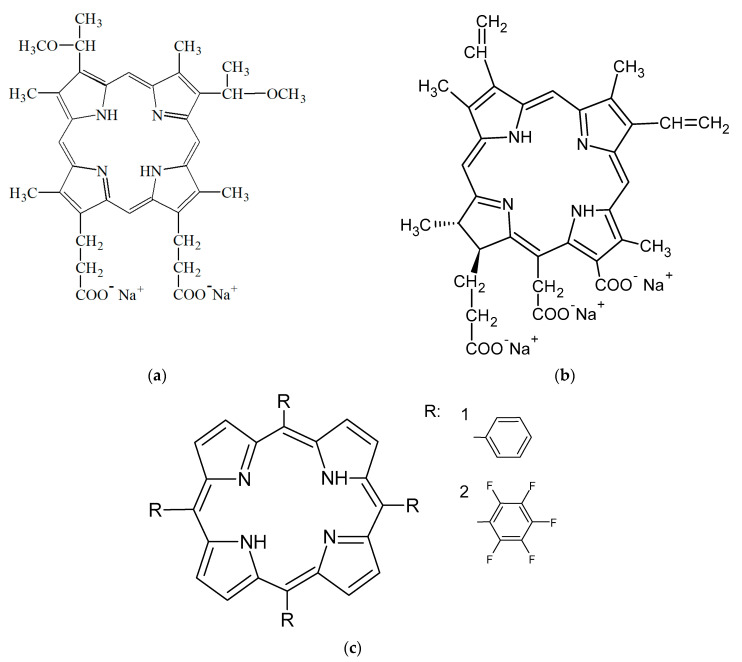
Structural formulas of porphyrins: disodium salt of 3,8-di (1-methoxyethyl) deuteroporphyrin IX (**a**), three sodium salt of chlorin e6 (**b**), 5,10,15,20-tetraphenylporphyrin (**c**, 1) fluorinated tetraphenyl porphyrin-5,10,15,20-tetrakis (pentafluorophenyl) porphyrin (**c**, 2).

Water-soluble porphyrins: disodium salt of 3,8-di (1-methoxyethyl) deuteroporphyrin IX (dimegin, DMG, synthesized by G.V. Ponomarev at the Institute of Biological and Medicinal Chemistry, Moscow, Russia) and three sodium salt of chlorin e6 (Ce6, Frontier scientific Inc., Logan, UT, USA), and hydrophobic porphyrins: 5,10,15,20-tetraphenylporphyrin (TPP, synthesized by G.V. Ponomarev at the Institute of Biological and Medicinal Chemistry, Moscow, Russia) and fluorinated tetraphenyl porphyrin-5,10,15,20-tetrakis (pentafluorophenyl) porphyrin (TPPF20, (Sigma-Aldrich, Saint Louis, MO, USA)) were used as photosensitizers. The structures of the photosensitizers are shown in Figure 1.

**Figure 2:**
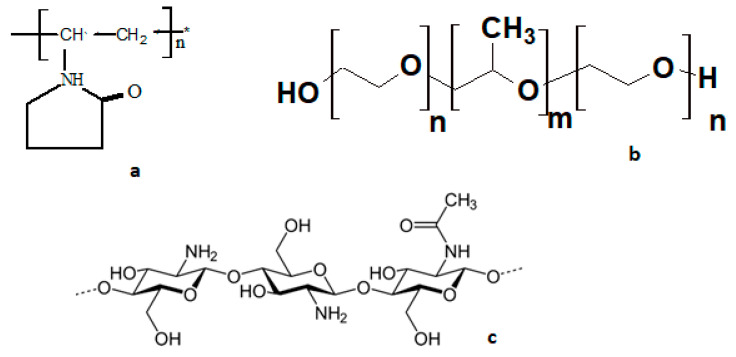
Structural formulas of polymers: poly-N-vinylpyrrolidone (**a**), ternary block copolymer of ethylene and propylene oxide, Pluronic F127, where n = 65; m = 200 (**b**), chitosan (**c**).

Poly-N-vinylpyrrolidone (PVP, 4 × 10^4^ M, Sigma-Aldrich, Saint Louis, MO, USA) and a ternary block copolymer of ethylene and propylene oxide, Pluronic F127 (F127, 12.6 × 10^−3^ M, BASF, Ludwigshafen, Germany), are well-studied and widely used in medicine. Chitosan (CT_100_) was purchased from Sigma-Aldrich (#448869) with a Mw = (50–190) × 10^3^ Da and a deacetylation degree DA = 75–85% and was used without additional purification (Sigma-Aldrich, Saint Louis, MO, USA). Chitosan was used as a biologically active polysaccharide, while tryptophan (Trp, Sigma-Aldrich, Saint Louis, MO, USA) was utilized as a substrate. The structures of polymers are shown in Figure 2.

### 2.2. Kinetics of Tryptophan Photo-Oxidation

**Figure 3:**
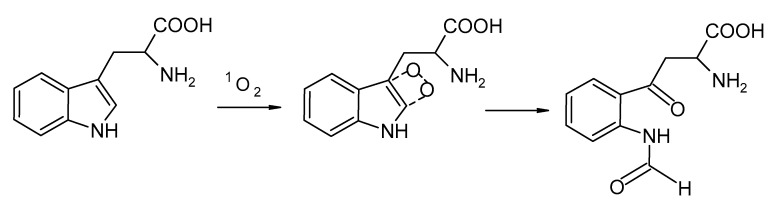
Reaction of oxidation of tryptophan with singlet oxygen.

The study of the activity of porphyrin-containing polymer systems during the generation of singlet oxygen in the aqueous phase was carried out using the reaction of oxidation of tryptophan with singlet oxygen with the formation of tryptophan endoperoxide (Figure 3).

Photosensitizing double systems (PS-AP and PS-CT_100_) with water-soluble PSs were prepared by mixing solutions of porphyrin and polymer or chitosan (in 0.2% acetic acid) for 10 min. [23]. To obtain ternary systems (PS-AP-CT_100_), porphyrin solutions were first mixed with an amphiphilic polymer for 10 min at room temperature, then a chitosan solution was added and mixed for another 10 min [23]. Solubilization of hydrophobic porphyrins was carried out according to the procedure described in [24]. The PS concentration is 5 × 10^−6^ M. The AP concentrations varied from 5 × 10^−6^ M to 5 × 10^−4^ M; the concentration of CT_100_ varied from 1 × 10^−4^ M to 6 × 10^−3^ M (given per unit).

Photo-oxidation of tryptophan (Trp) with molecular oxygen dissolved in water was carried out in a 1 cm thick quartz cell at room temperature. The concentration of tryptophan is 10^4^ M. The kinetics of the process was monitored by the change in concentration of tryptophan, which was determined from the optical density value of the absorption band (λ = 280 nm) in the UV spectrum of tryptophan.

For a comparative assessment of the activity of porphyrin-containing systems in the test reaction of photo-oxidation of tryptophan in an aqueous medium, the effective specific rate constant k_eff_, determined from the initial linear portion of the kinetic dependence C_i_ = C_i_ (t), was introduced:

k_eff_ = (1/t) × ln(C_0i_/C_i_)/C_PPS_ (1)

where C_0i_ is the initial concentration of substrate i, C_i_ (t) is the concentration of substrate I at the time t (s) of photo-oxidation, C_PPS_ is the concentration of photosensitizer. The measurement error in determining k_eff_ was 10%.

The UV spectra and electronic absorption spectra (EAS) of the solutions were recorded on a Cary50 spectrophotometer (Varian, Mulgrave, VIC, Australia); the fluorescence spectra were recorded on a Cary Eclipse spectrofluorimeter (Varian, Mulgrave, VIC, Australia).

### 2.3. Dynamic Light Scattering

The particle size and Zeta potential of polymers (AP and CT_100_) and TPPF20 solubilized by F127 in aqueous solutions in the initial state and in the ternary systems (TPPF20-F127-CT_100_) systems was determined by dynamic and electrophoretic light scattering on a Malvern Zetasizer Nano ZS (Malvern Instruments Ltd., Malvern, UK), equipped with a He-Ne laser (λ = 633 nm) at an angle of 173°. ξ-potential was measured with laser Doppler velocimetry by determining the electrophoretic mobility. The Henry equation was used for calculation of ξ-potential values. A dielectric constant of 78.5 and a viscosity of 0.8872 mPa·s for pure solvent were used for all systems taking into account the low polymer concentration. The solutions were filtered through a standard 0.22 μm membrane into an optical cell (1 × 1 cm^2^). Data were processed using Zetasizer Software 6.2 (Malvern Instruments Ltd., Malvern, UK).

### 2.4. X-ray Diffraction

X-ray diffraction study of polymeric films was carried out in the Institute of Biochemical Physics using X-ray diffractometer with position-sensitive detector of local design described elsewhere [25] (CuKα radiation, sample to detector distance 105 mm, the width of X-ray beam and detector window 4 mm). The intensity of X-ray scattering was measured in transmission geometry in the range of diffraction vector module 0.04 nm^−1^ < S < 4.5 nm^−1^ (S = 2sinθ/λ, 2θ—scattering angle, λ—X-ray wavelength, equal for CuKα radiation to 0.1542 nm). The films were obtained according to the method described in [26]; the mass ratio of the components in the CT_100_-PVP film was 1:2 and 1:5, while for the ternary system DMG-PVP-CT_100_ the same ratio was 1:5:10.

### 2.5. Atomic Force Microscopy

The study of the surface structure of polymer films by atomic force microscopy (AFM) was carried out on a MultiMode 8 atomic force microscope (Bruker, Santa Barbara, CA, USA) using an RTESPA-300 probe (Bruker, Santa Barbara, CA, USA). The surface topography of polymer films, as well as their local mechanical properties (Young’s modulus), were studied using the PeakForce QNM method. Sreas of 5 × 5 μm^2^ were investigated. The films were obtained according to the method described in [26]; the mass ratio of the components in the CT_100_-PVP film was 1:1 and 1:2.

## 3. Results and Discussion

### 3.1. Photocatalytic Activity of Water-Soluble PS

**Figure 4:**
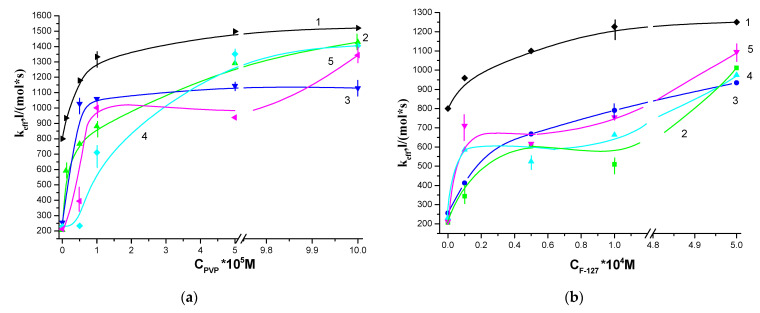

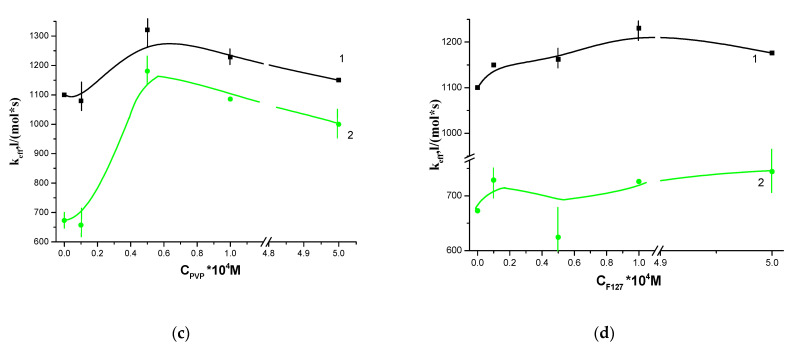
Dependence of the effective rate constant of tryptophan photo-oxidation, catalyzed by the ternary systems photosensitizers- amphiphilic polymers-chitosan (PS-AP-CT) on the concentration of AP, where PS = dimegin (DMG) (**a**,**b**), Ce6 (**c**,**d**). Where, AP-polyvinylpyrrolidone (PVP) (**a**,**c**), F127 (**b**,**d**). C_CT100_ = 0 (curve 1); C_CT100_ = 10^−4^ M (curve 2); C_CT100_ = 6 × 10^−4^ M (curve 3); C_CT100_ = 10^−3^ M (curve 4); C_CT100_ = 6 × 10^−3^ M (curve 5). The concentration of porphyrin is C = 5 × 10^−6^ M, the concentration of tryptophan is 10^−4^ M.

**Figure 5:**
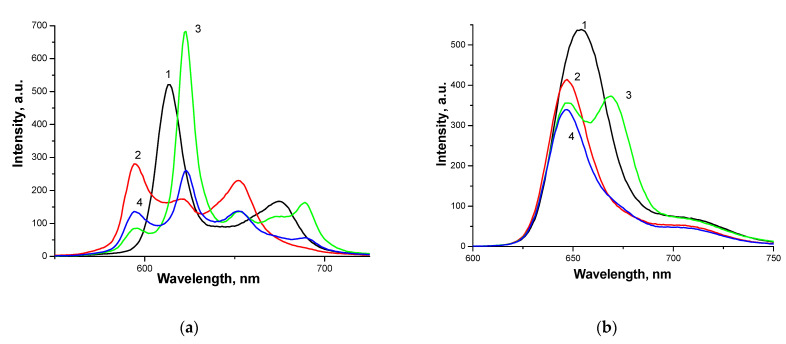
Fluorescence spectra for PS: DMG (**a**, curve 1) and Ce6 (**b**, curve 1), PS complexes with chitosan (curve 2) and for ternary systems: PS-PVP-CT_100_ (curve 3), PS-F127-CT_100_ (curve 4). The concentration of porphyrin is C = 5 × 10^−6^ M, C_CT100_ = 6 × 10^−4^ M, C_AP_ = 10^−4^ M.

As follows from Figure 4, the solubilization of porphyrins with Pluronic F127 and PVP made it possible to weaken the effect of CT_100_ on the photocatalytic activity of water-soluble porphyrins. It should be noted that in the presence of CT_100_, the photocatalytic activity of pure DMG and Ce6 decreases by a factor of 4 and 2, respectively (Figure 4a,b, zero points of curves 1 and 2–5, respectively). As the PVP content in the solution increased, the effective rate constant of tryptophan oxidation k_eff_ in the presence of DMG-CT_100_ increased (Figure 4a, curves 2–5). It is interesting to note that the concentration of chitosan had little effect on the values of k_eff_. Thus, with an increase in the concentration of PVP, the values of the effective constant k_eff_ at all concentrations of chitosan (Figure 4a) approach the values of k_eff_ for the corresponding binary system DMG-PVP. Thereby, when PVP is added to the DMG-CT_100_ system, a significant (4–5 times) increase in the effective constant k_eff_ of the photo-oxidation rate of tryptophan is observed (Figure 4a), which is apparently associated with the partial binding of porphyrin molecules, that were previously localized in the aggregated state near the amino groups of chitosan, with polyvinylpyrrolidone macromolecules. From the literature it follows that the supramolecular structure of chitosan in solutions, which possibly determines the functional activity of the polysaccharide, depends on its molecular weight and the degree of deacetylation [27,28]. Since the values of the degree of deacetylation for CT_20_ and CT_100_ are close, it is obvious that the conformation of chitosan under the conditions of our experiments is determined by its molecular weight. According to [29] low molecular weight chitosan in weakly acidic solutions is present both in the form of aggregates of macromolecules, in which amino groups (responsible for PS binding) are involved in the formation of intermolecular hydrogen bonds, and in the form of free macromolecules capable of interacting with PS. Such interaction, according to [22], when using chitosan with M = 20 kDa, cannot be completely eliminated even in the presence of high concentrations of PVP. The supramolecular structure of chitosan with higher molecular weight in weakly acidic solutions is a flexible worm-like chain [28,30]. This structure is supported by electrostatic, hydrogen and hydrophobic bonds, in which amino groups are involved. In this case, the addition of PVP to the DMG-CT_100_ system makes it possible to almost completely eliminate the interaction of porphyrin with CT_100_ and to increase the photocatalytic activity of the system. This is indicated, among other things, by an increase in the intensity of all fluorescence bands in the spectra of the DMG-CT_100_ systems upon the addition of PVP (Figure 5a).

As seen from Figure 4b, the solubilization of DMG with Pluronic F127 is less effective and does not completely prevent the interaction of CT_100_ with porphyrin in the DMG-F127-CT_100_ system. When Pluronic is used as an AP, the activity of the DMG-F127-CT_100_ system increases slightly (Figure 4b) above the level of free DMG. This difference is primarily due to the presence of 0.2% acetic acid (a solvent of chitosan) in the solution, which affects the supramolecular structure of F127 in solution. It is important to note here that the supramolecular structure of PVP is practically independent of the pH of the medium [31]. We have shown that the photocatalytic activity of DMG-F127 complexes in acetic acid drops sharply [32]. This effect is associated with the aggregation of Pluronic micelles in an acidic medium [33], which leads to an increase in the size of polymer associates and compaction of micelles. It can be assumed that as a result of such processes, the formation of pseudo-polycation of Pluronic occurs, which leads to the aggregation of PS and a decrease in the values of the effective rate constant k_eff_ of substrate oxidation.

For Ce6-CT_100_, a similar dependence of the effective rate constant of tryptophan oxidation k_eff_ on the PVP concentration was obtained, which takes the form of curves with a maximum at a PVP concentration of 5 × 10^−5^ M (Figure 4c, curve 2) and is similar to the dependence in the absence of CT_100_ (Figure 4c, curve 1). In this case, the addition of PVP to the Ce6-CT_100_ system also increases the photocatalytic activity, but the k_eff_ values do not reach the k_eff_ values of the Ce6-PVP binary system. It is interesting to note that the addition of Pluronic F127 to the Ce6-CT_100_ system does not change its photocatalytic activity (Figure 4d, curve 2), which is due to the fact that Ce6 is a more hydrophilic porphyrin than DMG and is localized at the edge of the F127 micelle [19] and, apparently, even in the presence of Pluronic, it is prone to aggregation near the amino groups of chitosan. Aggregation is indicated, among other things, by a decrease in the intensity of the band in fluorescence spectra of the Ce6-CT_100_ systems upon the addition of F127 (Figure 5b, curve 4).

### 3.2. Photocatalytic Properties of Hydrophobic PS

**Figure 6:**
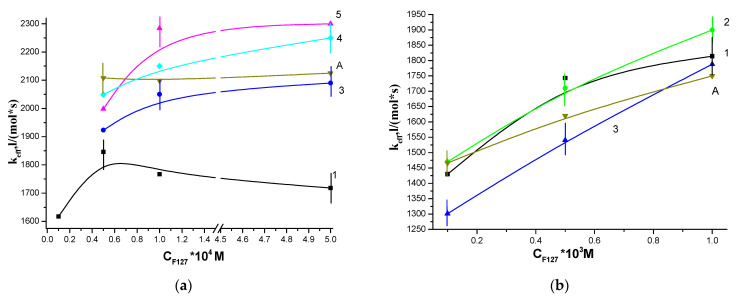
Dependence of the effective rate constant of tryptophan photo-oxidation catalyzed by the PS-F127-CT_100_ ternary systems on the concentration of F127, where PS = TPPF20 (**a**), tetraphenylporphyrin (TPP) (**b**). Where, C_CT100_ = 0, C_CH3COOH_ = 0.2% (curve A); C_CT100_ = 0 (curve 1); C_CT100_ = 10^−4^ M (curve 2); C_CT100_ = 6 × 10^−4^ M (curve 3); C_CT100_ = 10^−3^ M (curve 4); C_CT100_ = 3 × 10^−3^ M (curve 5). The concentration of porphyrin C = 5 × 10^−6^ M, the concentration of tryptophan is 10^−4^ M.

We have previously shown that solubilization of TPP and its analogs with amphiphilic polymers (AP), such as pluronics (ternary block copolymers of ethylene oxide and propylene oxide), polyethylene oxide and PVP, makes it possible to obtain effective water-soluble photosensitizers for the generation of singlet oxygen [24]. Figure 6 demonstrates the dependences of k_eff_ of the photo-oxidation rate of tryptophan in the presence of TPPF20 (a) and TPP (b), solubilized by Pluronic and the dependence of their ternary systems with chitosan on the concentration of Pluronic F127. It can be seen that in the case of TPPF20-F127 systems, the addition of chitosan (Figure 6a, curves 2–4) leads to an increase in photocatalytic activity by a factor of 1.1–1.3 compared to the activity of TPPF20 solubilized by Pluronic F127 in acidic or neutral media. This growth is primarily associated with the effect of acetic acid on the activity of porphyrins (see curve 1 (neutral medium) and curve A (0.2% CH_3_COOH), Figure 6a). In the case of hydrophobic TPP solubilized with F127, k_eff_ does not change neither in the presence of CT_100_ (Figure 6b, curves 2–4) nor in the presence of acid (Figure 6b, curve A).

**Figure 7:**
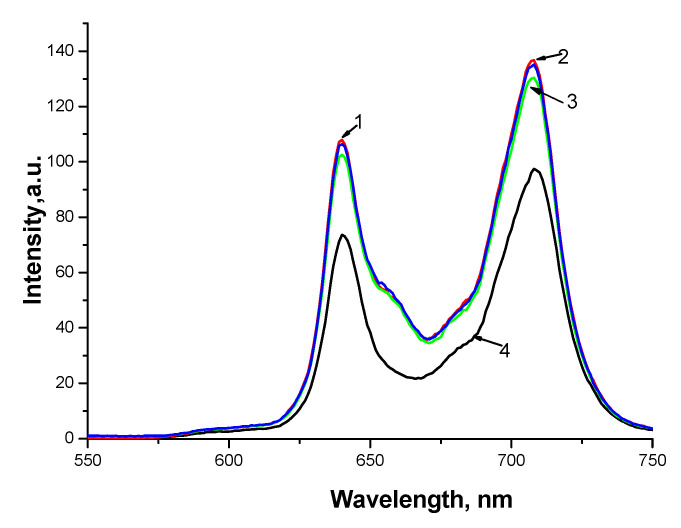
Fluorescence spectra for TPPF20-F127 in water (1, red), in the presence of chitosan (2, blue) and acetic acid 0.2% (3, green), in chloroform (4, black). The concentrations: C_TPPF20_ = 5 × 10^−6^ M, C_CT100_ = 6 × 10^−4^ M, C_F127_ = 10^−4^ M.

Such differences in the photosensitizing properties of hydrophobic PSs can be associated with different degrees of aggregation of solubilized tetraphenylporphyrins. Thus, for solubilized TPPF20, the maximum activity is achieved at F127 concentration of 5 × 10^−5^ M (Figure 6a, curve 1), while for solubilized TPP, this happens at Pluronic concentrations of 5 × 10^−4^ M–1 × 10^−3^ M (Figure 6b, curve 1). Thus, TPPF20 is less aggregated and therefore more evenly distributed over Pluronic micelles. Apparently, the reason for the lower aggregation of TPPF20 during solubilization is the presence of fluorine atoms, which provide better binding to Pluronic. According to Bin Yang et al., in case of Pluronic, low pH values of the medium cause aggregation of F127 micelles, i.e., micelles “stick together” through the bridges from protonated water molecules, which leads to an increase in the size of polymer associates and to the compaction of micelles [33]. In this case, such “sticking” leads to the effective concentration of TPPF20 and the substrate within the sticky micelles and a certain increase in activity. Whereas in the case of TPP (less disaggregated in comparison with TPPF20), the interaction of micelles does not lead to effective concentration of PS. On the contrary, when PS-unfilled micelles “stick together”, the substrate is spatially distant from PS, which is observed at high AP concentrations. The invariability of the state of PS themselves is confirmed by the unchanged fluorescence spectra of solubilized tetraphenylporphyrins upon addition of chitosan (Figure 7).

### 3.3. Methods for Studying Supramolecular Interactions in the PS-AP-CT_100_ System

To predict the nature of interactions between the components of the PS-AP-CT_100_ system in solution, ξ-potentials of individual F127 and CT_100_, as well as TPPF20 solubilized by F127, in the absence and presence of CT_100_ were measured. Table 1 demonstrates that F127 in a neutral solution is characterized by a low negative value of the ξ-potential (−8 mV). In an acetic acid solution, the ξ-potential of F127 remains low; however, its sign changes to the opposite (+5 mV), which confirms the formation of the pseudo-polycation of Pluronic micelles in acetic acid. Chitosan molecules with a high degree of deacetylation in an acidic medium are characterized by a high positive charge due to protonation of amino groups (+53 mV). Micelles TPPF20-F127 have a small positive charge (+4 mV), which practically corresponds to the charge of F127 in an acidic solution. In the ternary system TPPF20-F127-CT_100_, the value of the ξ-potential is +36 mV, which is significantly lower than that of free chitosan. This effect may indicate the interaction of chitosan with TPPF20+F127 micelles (1%). It is unlikely that this interaction is of an electrostatic nature, since the components have the same charge. It is most likely that the interaction is of a hydrophobic nature (the available hydrophobic surface of the chitosan molecule is ≈50%), and the decrease in surface charge of the interaction product is due to the rearrangement of the surface of micelle.

**Figure 8:**
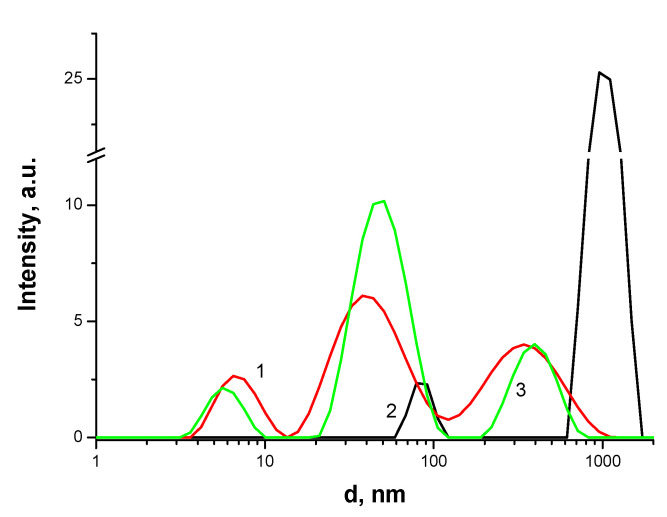
Size distribution of associates of TPPF20-F127 (1), CT_100_ (2), TPPF20-F127-CT_100_ (3) in aqueous and acetic acid solutions. The concentration of porphyrin C = 5 × 10^−6^ M, the concentration of CT_100_ is 0.05 mass% and concentration of F127 is 1 mass%.

As can be seen from Figure 8, for TPPF20 solubilized with F127 in the presence of CT_100_, there is a change in the characteristic sizes of porphyrin associates (Table 1, line 9, 10). As shown above, such changes can be associated with the interaction of Pluronic micelles with chitosan macromolecules. It is obvious that the presence of hydrophobic PSs in Pluronic micelles promotes the interaction of micelles with chitosan macromolecules, most likely due to hydrophobic interactions.

The size of chitosan associates in a 0.2% acetic acid solution in the presence and absence of AP and PS was also studied by the method of dynamic light scattering (Table 1). It was found that the sizes of AP associates in a joint solution of AP and CT_100_ remain unchanged (Table 1, line 1, 6, 7 and line 4, 6, 8). In this case, the size of CT_100_ associates increases in a joint solution with AP, which is associated with the “salting out” effect. From the data obtained, it follows that the interactions between AP and CT_100_ in a 0.2% acetic acid solution are negligible.

**Figure 9:**
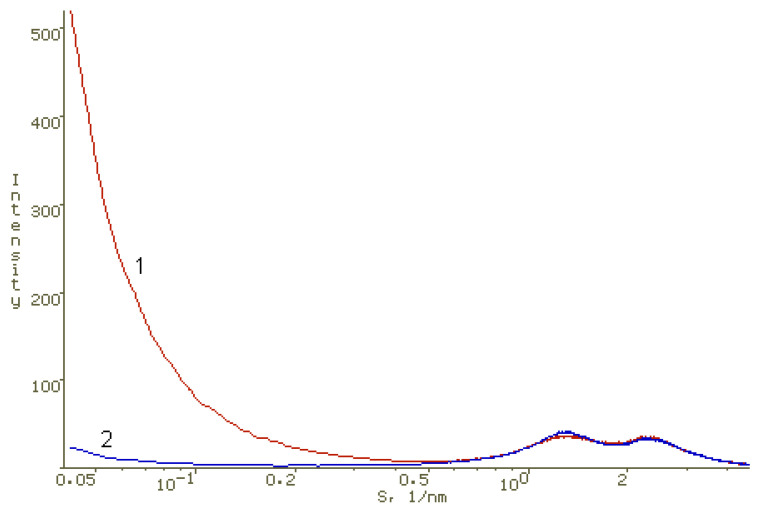
Experimental X-ray diffraction pattern of a chitosan-PVP film (1) with a mass ratio of these polymers of 1:2 and a model X-ray diffraction pattern of such film (2) obtained by summation of X-ray scattering intensity of chitosan and PVP films taken in a 1:2 proportion. Logarithmic scale along the abscissa axis.

**Figure 10:**
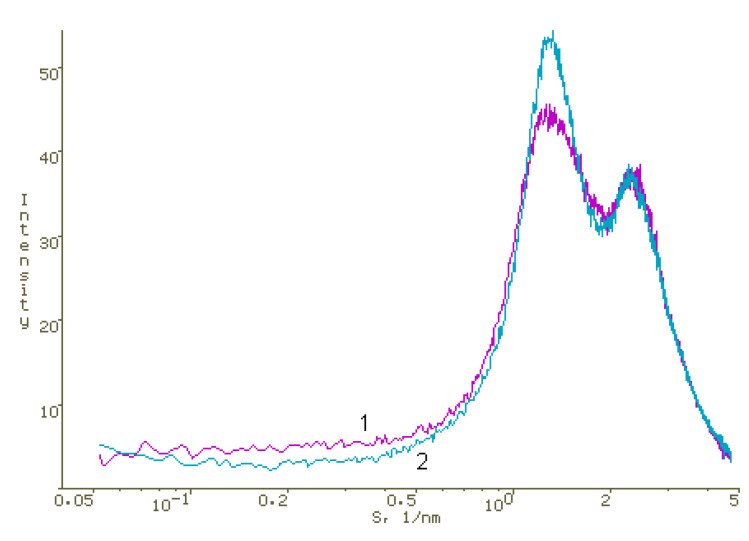
Diffraction pattern of the DMG-PVP film (1:10) (1) and the PVP film (2). Logarithmic scale along the abscissa axis.

To identify interactions in PS-AP-CT_100_ systems, where water-soluble dimegin acted as PS, AFM and XRD studies were carried out. The methods of preparing films for XRD and AFM studies are given in the Materials and Methods section. X-ray diffraction data indicate that chitosan and PVP in films obtained by evaporation of acetic acid aqueous solutions containing both of these polymers form two separate polymer phases (Figure 9) and DMG can be localized in significant amounts in a finely dispersed form in the PVP phase, leading to small changes in the structure of PVP (Figure 10).

**Figure 11:**
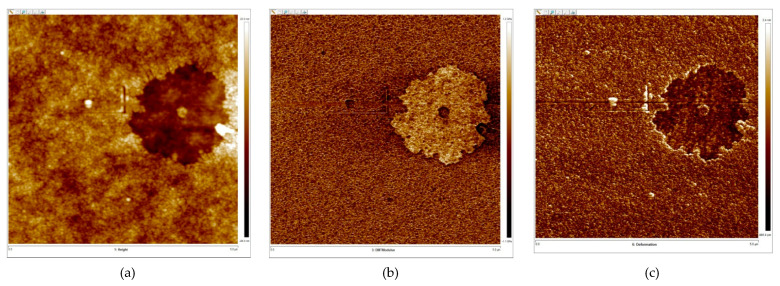
AFM images of the surface area of the CT100-PVP film obtained by evaporation of the solution (at a mass ratio of polymers 1:2) in topography mode (**a**), Young’s modulus (**b**) and deformation (**c**) measured in the PeakForce QNM mode.

The presence of two polymer phases in films obtained from joint solutions of chitosan and PVP was also confirmed by the AFM method. The local mechanical characteristics (deformation, Young’s modulus and adhesion) of films of a mixture of chitosan polymers and PVP with a thickness of 100 nm were measured in 5 × 5 μm^2^ areas in the PeakForce QNM mode. Microinhomogeneities are formed on the surface of thick films of binary mixtures, and phase separation is observed on a nanoscale (Figure 11). It turned out that the local mechanical characteristics of the film surface in microinhomogeneities differ, which indicates the presence of two phases. Young’s modulus value for the soft phase was 1.8 GPa, while for the harder phase it was 2.4 GPa. For the film obtained from the acetic acid solution of chitosan, Young’s modulus was 2.4 GPa, which corresponds to the value of Young’s modulus of the harder phase of the polymer mixture film.

## 4. Conclusions

It was shown that photosensitizing systems based on water-soluble porphyrins and polyvinylpyrrolidone in the presence of chitosan (molecular weight ~100 kDa) exhibit high photocatalytic activity, reaching the corresponding values observed for binary PS-AP systems. Hydrophobic porphyrins (TPP and TPPF20), solubilized by Pluronic F127, showed a higher efficiency in the generation of singlet oxygen (in the process of photo-oxidation of tryptophan) in the presence of chitosan (Mw ~100 kDa), than in its absence. According to AFM and X-ray data, chitosan and amphiphilic polymer form separate phases in solid films. Different methods, such as DLS in solution, AFM and XRD in solid films confirmed that CT_100_ does not interact with amphiphilic polymers F127 and PVP. At the same time, according to X-ray diffraction data for the DMG-PVP- CT_100_ systems, dimegin was localized in a finely dispersed state in the PVP phase.

## Figures and Tables

**Table 1:** Size distribution of associates and ξ-potentials in aqueous and acetic acid solutions of CT_100_, AP, CT_100_-AP, TPPF20-F127, TPPF20-F127-CT_100_.

№	Solution Composition	d, nm			ξ-Potential, mV
		Peak1	Peak2	Peak3	
1	F127 (2 mass%)	6.9	56		
2	F127 (1 mass%)	7.2	62		−8
3	F127 (1 mass%) + CH_3_COOH (1 vol.%)	7.6	53	396	+5
4	PVP (1 mass%)	9	165		
5	CT_100_ (0.05 mass%)		89	960	+53
6	CT_100_ (0.2 mass%)		112	790	
7	CT_100_ (0.2 mass%)-F127 (2 mass%)	6.8	50	1250	
8	CT_100_ (0.2 mass%)-PVP (1 mass%)	10.4	154	1320	
9	TPPF20-F127(1 mass%)	6.6	46	490	+4
10	TPPF20-F127(1 mass%)-CT_100_ (0.05 mass%)	8	50	405	+36

The relative measurement error was no more than 5%.

## Data Availability

The data presented in this study are available on request from the corresponding author.
